# Nowhere to go: disclosure and help-seeking behaviors for survivors of violence against women and girls in South Sudan

**DOI:** 10.1186/s13031-020-0257-2

**Published:** 2020-02-12

**Authors:** Maureen Murphy, Mary Ellsberg, Manuel Contreras-Urbina

**Affiliations:** The Global Women’s Institute at the George Washington University, 2140 G Street NW, Washington DC, 20052 USA

**Keywords:** Violence against women and girls, Conflict-affected settings, Intimate partner violence, Non-partner sexual violence, Help seeking behaviours

## Abstract

**Background:**

Despite high rates of violence against women and girls (VAWG) in conflict and humanitarian contexts, many survivors do not tell anyone about their experience or seek help from support r services (e.g. health, legal, psychosocial support, police).

**Methods:**

This paper examines disclosure and help seeking behaviours of survivors of non-partner sexual violence (NPSV) and intimate partner violence (IPV) among women and girls aged 15–64 from three sites in South Sudan. It seeks to understand how exposure to armed conflict is associated with disclosure and help seeking practices.

**Results:**

For NPSV, respondents for whom an incident of sexual violence occurred during conflict had twice the odds of telling someone about their experience (aOR: 2.2; 95%CI: 1.3–3.7; *p* < 0.01) and three times the odds of seeking help (aOR: 3.1; 95%CI: 1.7–5.9, *p* < .001), compared to respondents for whom the incident of violence did not occur during conflict. Age, the identity of the perpetrator, working status of the woman, poverty and location also affected disclosure and help seeking behaviours for survivors of NPSV. For IPV, exposure to conflict increased the odds a respondent would tell someone about her experience (aOR 1.7; 95%CI 1.2–2.5; *p* < .01), but was not associated with seeking support services. The severity of IPV affected both disclosure and help seeking behaviours, with the odds of disclosing IPV increasing if the respondent experienced both physical and sexual IPV (compared to only sexual violence), had been injured, thought their well-being was affected, was afraid of their partner, or was controlled by their partner. However, not all these factors were subsequently associated with help seeking behaviours for survivors of IPV and respondents who reported they were sometimes afraid of their partner had reduced odds of seeking help, compared to those who were never afraid of their partners.

**Conclusions:**

These findings are important as, prior to this analysis, it was unclear how experiencing conflict-related VAWG would influence disclosure and help seeking. Given the findings of this paper, it is important that the international community consider how to reduce barriers to reporting and help seeking for non-conflict-related forms of violence in these settings.

## Background

Violence against women and girls (VAWG) is a pervasive problem during times of armed conflict. Global estimates show that approximately 21.4% of women and girls in complex humanitarian emergencies have experienced sexual violence at least once during their lifetimes [22]. This rate is approximately three times greater than estimated rates of sexual violence against women and girls globally (7.2% over the course of their lifetimes) – suggesting that exposure to armed conflict has a considerable effect on increasing incidents of sexual violence [24]. However, sexual violence is not the only form of VAWG to affect the lives of women and girls in conflict settings. In fact, more women and girls are reported to experience intimate partner violence (IPV) compared to non-partner sexual violence (NPSV) even during armed conflict [15]. Exposure to armed conflict is also emerging as a risk factor for increased IPV in conflict and post-conflict contexts [8, 11, 18].

Despite the high rates of VAWG in conflict and humanitarian contexts, many women and girls do not seek help through support services (e.g. health, legal, psychosocial support, police) after an experience of violence [1]. Previous research in non-conflict settings has shown that the factors preventing access to support services are complex and multifaceted. While this research has shown that women and girls often tell someone about the violence they are experiencing, this disclosure does not necessarily mean survivor’s access support services [2, 13, 23]. Some of the barriers that prevent access to these services include: a lack of services, a lack of trust in the services that are available, cultural norms that view experiences of VAWG as normal, gender inequality, stigma, a lack of trained or committed service providers, and lack of independent financial resources [10, 13, 19]. Experiencing more severe violence and independent access to financial resources or capital has been seen to promote access to support services in non-conflict settings [2, 3, 23].

Survivor support services may be even more inaccessible in contexts affected by armed conflict. In many cases, conflict can prevent the establishment of these services or interrupt previously existing service provision. When support services are available, they may be of poor quality, lack the necessary drugs and supplies and be staffed by personnel who have not been trained to provide supportive and non-judgmental services to survivors [6, 7, 21]. In addition, armed conflict can cause women and girls to lose their previous social support networks and increase economic insecurity, which may also influence disclosure and help seeking behaviours [9, 18].

The purpose of this paper is to increase our understanding of how exposure to armed conflict is associated with disclosure and help seeking behaviours for survivors of VAWG. While previous research has examined disclosure and help seeking behaviours in non-conflict settings, there has been no research on how violence experienced during armed conflict is associated with these behaviours.

This research specifically focuses on the experiences of women and girls in South Sudan, a context that has experienced almost continuous armed conflict both before and after it became an independent country in 2011. Two consecutive civil wars (one from 1955 to 1972 and the second from 1983 to 2005) heralded its independence from Sudan in 2011. After the signing of the peace agreement in 2005, the country experienced relative peace until December 2013 when large-scale violence broke out in Juba and caused more than one million people to flee their homes and tens of thousands to seek shelter on United Nations (UN) bases – termed Protection of Civilian (PoC) sites - throughout the country [20].

Women and girls in South Sudan experience violence both as a result of these ongoing conflicts as well as due to patriarchal norms and pervasive gender-inequality in the country. Recent research has found that more than half of women and girls from three sites surveyed in South Sudan have experienced partner or non-partner violence in their lifetime; however, women and girls lack access to basic live-saving gender-based violence (GBV) services throughout the country (Gender-based Violence Sub -[5, 18]). Within this context, there are numerous socio-cultural factors that may reduce a survivor’s willingness to disclose experiences of violence and seek support. For example, women and girls often accept VAWG as normal [14, 18]. Furthermore, conventions on divorce require women to pay back any bride price their family paid, creating a financial burden that many women cannot meet, while men typically keep custody of the children if a marriage dissolves [16].

For un-married survivors of NPSV, if their loss of virginity becomes known it can affect their ability to marry and assume the expected societal role of wife and mother [11]. In addition, the traditional redress after the rape of an unmarried girl is a marriage between the survivor and the perpetrator of the violence [17]. These consequences may potentially reduce incentives to disclose violence and seek help for unmarried survivors of NPSV. In addition, the limited availability of support services for survivors, and the insistence of many health care providers that survivors produce a police report before providing services are also considerable barriers to disclosure and service access for survivors of VAWG in South Sudan [12, 18].

Within this wider context of gender inequality, patriarchal norms and limited services, the specific impact of armed conflict on disclosure and reporting rates is not known. This paper will seek to bridge this gap and explore how exposure to armed conflict is associated with disclosure and help seeking behaviours for women and girl survivors of violence in South Sudan.

## Methods

### Data and study population

This paper examines the results of a cross-sectional survey undertaken amongst women and girls aged 15–64 from three purposively selected sites (Juba City, Rumbek and the Juba Protection of Civilian Sites) in South Sudan in 2016. A total of 2244 women and girls were interviewed, with an overall response rate of 84%. Of these, 1359 had experienced IPV or NPSV and were included in this sub analysis. The data was collected as part of the research programme of the *What Works to Prevent Violence Against Women and Girls in Conflict and Humanitarian Crises* Consortium. Study design and data collection was led by the Global Women’s Institute (GWI) at the George Washington University and the International Rescue Committee (IRC) with support from CARE International UK and Forcier Consulting.

Households were selected through a multi-stage cluster sampling process where bomas (typically the size of a neighbourhood) or blocks in the PoC sites were first randomly selected. Individual households were then selected through a systematic sample with a random starting point within each selected cluster. At each household, all eligible women and girls who were resident were listed and a final respondent randomly selected by a random number generator. Further information on the sample and sampling strategy employed in the study can be found in previously published articles and reports [11, 18].

### Study sites

The study was implemented in three diverse sites. The first was Juba City, which is the capital of South Sudan and the country’s largest population centre. It was affected by the 2013 Crisis where there was active conflict within the city centre as well as surrounding areas. Despite this, compared to other locations in South Sudan, Juba is one of the most developed areas in the country and has considerably more services of survivors of VAWG and access to government systems as well as international aid. Secondly, the study was implemented in Rumbek in Lakes State. The population in this location primarily depends on a pastoralist lifestyle and cattle raids and other inter-communal conflicts are common. At the time of this study, this site had an international NGO providing GBV case management services. Finally, the study was conducted in Juba PoCs, which were created in response to ethnic and political conflict that began in 2013. Almost 40,000 people were resident in the PoCs during the study period and relied almost entirely on the international community for services [20]. At the time of the study, multiple international NGOs were providing GBV services in the sites.

### Primary measures

The survey was based on the *WHO Multi-country Study on Women’s Health and Domestic Violence Against Women* [4]*.* This paper focuses specifically on disclosure and help seeking behaviours for survivors of NPSV and physical or sexual IPV. The study focused on a combined measure of physical or sexual IPV, while only examining sexual non-partner violence for a number of reasons. Primarily, these measures were chosen so the results would be applicable to practitioners who see cases of both physical or sexual IPV but, for non-partner violence, often focus on forms of sexual violence. Furthermore, in a conflict-context it may be difficult to delineate between “gendered” non-partner physical violence and general exposure to armed-conflict, making it difficult to examine both physical and sexual non-partner violence as a form of gender-based violence. A respondent’s status as a survivor of each of these forms of violence was assessed through a series of yes/no questions about act-based experiences of violence (e.g. beaten, hit, forced to have sex when she did not want to, etc.). See previously published articles from this study for more information on how a respondent’s survivor status was determined [11, 18].

Disclosure and help seeking behaviours were measured separately for NPSV and IPV. Respondents were first asked if they told anyone about their experience of IPV or NPSV (as relevant based on their survivor status). After asking about disclosure, separate questions were asked about where the respondent sought help after the experience of violence. These questions were also asked separately for IPV and NPSV survivors. The responses for each series of questions were combined into a binary yes/no variable for analysis. For disclosure, this resulted in a binary variable “yes they told someone/no they told no one”. For help seeking behaviours, accessing a service was defined as accessing help from any of the following: police, health service provider, local or formal court, local leader/chief, religious leader, women’s organization, the United Nations (UN) or a non-governmental organization. This was also coded as a binary variable “yes sought help from a support service/no did not seek help from a support service” for analysis. Secondary analysis was also undertaken comparing those who sought help from more “formal” services (police, health, formal courts or NGOs/UN) compared to “informal or community-based services” (local courts, local leaders, religious leaders, women’s organizations). Respondents who accessed both formal and informal services were classified as utilizing formal services.

Exposure to armed conflict was also measured separately for NPSV and IPV. For NPSV, after disclosing an incident of sexual violence (SV), the respondent was asked if this experience happened during: intercommunal violence, an abduction, or while being displaced. If the respondent answered yes to any of these variables, then the response was coded as having experienced conflict-related sexual violence. For IPV, conflict-exposure was assessed through a question about if the respondent had ever been seriously injured, physically disfigured, or abducted or if she had ever experienced an attack on her village/community of residence. This was coded into a binary variable reflecting “yes-exposed to conflict/no- not exposed to conflict”.

Other potential factors that may affect disclosure and help seeking behaviour were also identified from the literature base and included in the analysis. For NPSV, this included the type of violence experienced (rape or another form of sexual violence such as unwanted touching), the identity of the perpetrator (an armed actor/policeman or someone else) and agreement about an attitude question assessing acceptance of NPSV *(“If a woman is raped, she has usually done something careless to put herself in that position”).*

For IPV, the type of violence experienced (sexual violence only, physical violence only or physical and sexual violence) was also examined. In addition, a partners use of controlling behaviours on his partner (a series of four questions about if the partner gets jealous or angry when she talks to another man, frequently accuses her of being unfaithful, forces her to ask permission before seeking healthcare for her or her children; or forces her to ask permission to move outside the house) was coded as a binary variable (yes/no). If a respondent had been injured due to the IPV (yes/no), self-reported impact of the IPV on wellbeing (no effect, a little effect, a large effect), and if the respondent was afraid of her partner (never, sometimes, most/all of the time) was also examined. Finally, respondents were asked if a husband was justified in beating his wife if she: goes out without telling him, neglects the children, argues with him, or refuses to have sex with him. If the respondent said yes to any of these reasons, the respondent was classified as agreeing that violence was justified (yes/no).

Further covariates such as location (Juba, Rumbek or the Juba PoCs), age (15–19, 20–29, 30–39, 40–64), marital circumstances (for IPV outcomes), main income source (no income or income from humanitarian aid, income from own work, income from husband’s work and other source of income), occupation (not working, student, working) and main source of cooking fuel (charcoal or other (wood, grass, leaves, etc.)) were controlled for in the final models.

### Data analysis

Quantitative data analysis was conducted using STATA Version 15.1. Descriptive statistics as well as bivariate (Pearson chi-square) and multivariate (logistic regression) analysis were utilized. For the analysis of factors related to disclosure of violence and help seeking behaviours, a multi-step process was utilized. First, individual relationships between the potential factors associated with disclosure and help seeking (for both IPV and NPSV separately) were explored using Pearson chi-square. Next, the independent variables and further covariates were separated into blocks (conflict exposure, violence disclosure factors and socio-demographics) and a final model was developed including all identified conflict exposure and violence disclosure factors as well as retaining the socio-demographic and marital circumstances (for IPV outcomes) variables that maintained significance in the final models. Standard errors were clustered at the boma/block level. Sampling weights were not used for this analysis. This was decision was made due to the potential of introducing bias given the fluctuating underlying population during the survey period due to ongoing conflict-related and economic migration as well as outdated census figures. In order to understand the effect this decision had on the results, the study team re-ran all the analysis employing sampling weights and did not find statistically different results than those presented in this paper.

## Results

Overall, there were 1359 women and girls who had experienced either IPV or NPSV and were included in the sample. About a quarter of the respondents were resident in Juba, while the remaining three-quarters were split between Rumbek and Juba PoCs. The sample was young (17% between 15 and 19 and 46% between 20 and 29) and a vast majority (81%) were currently or had been married. A majority were not working or a student (64%) and almost 30% had no income or were completely dependent on humanitarian aid. See Table 1 for more details.

**Table 1:** Socio-demographics of women and girls who experienced NPSV or IPV and disclosed the experience or sought help

	Overall Sample (*n* = 1359)	NPSV disclosure and help seeking (*n* = 779) % (95%CI)		IPV disclosure and help seeking (*n* = 1163) % (95%CI)	
		Told Someone	Sought Help	Told Someone	Sought Help
Total		54% (50–58)	27% (23–30)	56% (53–59)	36% (33–38)
Location					
Juba	24%	49% (42–57)***	11% (7–17)***	49% (43–55)***	15% (11–20)***
Rumbek	39%	64% (58–69)***	31% (26–37)***	70% (66–74)***	52% (48–57)***
Juba PoCs	37%	48% (43–54)***	30%(25–35)***	42% (37–47)***	29% (25–34)***
Age					
15–19	17%	48% (41–55)	22% (17–29)	42% (34–51)***	26% (19–35)**
20–29	46%	53% (48–58)	25% (21–30)	53% (49–57)***	33% (30–37)**
30–39	22%	58% (50–65)	27% (30–35)	61% (55–67)***	37% (32–43)**
40–64	14%	64% (54–74)	38% (28–49)	64% (57–71)***	46% (39–53)**
Education					
No education	44%	55% (49–60)	31% (26–36)*	63% (59–67)***	46% (42–50)***
Primary education	28%	53% (47–59)	23% (18–29)*	48% (43–54)***	27% (22–32)***
Secondary or higher	28%	54% (48–61)	23% (18–29)*	50% (45–56)***	26% (21–31)***
Occupation					
Not Working	64%	52% (47–56)	27% (23–31)*	57% (54–61)**	36% (33–39)
Student	14%	54% (46–63)	18% (13–26)*	41% (32–50)**	26% (19–35)
Working	23%	62% (54–69)	32% (25–40)*	58% (52–63)**	38% (33–44)
Primary Source of Income					
No Income/ Humanitarian Aid	28%	46% (40–52)**	30% (25–36)	48% (42–53)**	34% (29–39)***
Money from own work	20%	63% (54–70)**	24% (18–32)	65% (59–70)**	50% (43–56)***
Support from husband	31%	54% (47–61)**	27% (21–33)	58% (53–62)**	30% (26–35)***
Other Support	21%	58% (51–65)**	23% (17–29)	53% (46–60)**	31% (25–38)***
Main Source of Cooking Fuel					
Wood/Gras/Other	41%	51% (46–57)	24% (19–29)	62% (57–66)***	45% (40–49)***
Charcoal	59%	56% (51–60)	28% (24–32)	51% (47–55)***	29% (26–32)***
House					
Hut [Tukul]	38%	57% (51–63)*	22% (18–27)	62% (58–66)***	42% (37–46)**
House/apartment	16%	61% (52–70)*	27% (19–36)	59% (52–66)***	30% (24–37)**
Temporary or communal shelter	45%	50% (45–55)*	29% (25–34)	48% (44–53)***	32% (28–36)**
Marital Status- Ever married	81%	55% (51–59)	29%* (25–32)	58% (44–61)***	38% (35–41)***

Note: * p < 0.05, ** p < 0.01, *** p < 0.001

For respondents who had experienced NPSV, 54% told someone about their experience and 27% sought help after their experience. There were statistically significant differences by location with respondents from Rumbek (64%) more likely to have told someone about their experiences compared to those from Juba (49%) and the Juba PoCs (48%) (*p* < .001). Respondents from Juba (11%) were also less likely to seek help compared to those from Rumbek (31%) and the Juba PoCs (30%) (*p* < .001). There were also significant differences with those who were ever married (29%) and were working (32%) were more likely to have sought help compared to those who were never married and were not working or were a student (*p* < 0.05). Finally, there were significant differences by income source with those who had no income or depended on humanitarian aid the least likely to tell someone about their experience of violence (46%) (*p* < 0.01). See Table 1 for more details.

For respondents who had experienced IPV, 56% had told someone about their experience while 36% sought help. There were statistically significant differences on almost all variables. For example, respondents from Rumbek more often told someone about their experience (70%) and sought help (52%) compared to those from Juba (49 and 15% respectively) and the Juba PoCs (42 and 29% respectively) (*p* < 0.001). Age was also important with older respondents more likely to tell someone (*p* < 0.001) and access services (*p* < 0.01). Respondents whose main source of income was money from their own work were more likely to tell someone (*p* < = 0.01) and seek services (*p* < =0.001) compared to those with other income sources.

### Conflict-exposure

For NPSV, respondents who had experienced conflict-related SV were significantly more likely to have told someone about their experience of violence (60%) and have sought help (36%) compared to those who hadn’t experienced conflict-related violence (*p* < 0.001). For IPV, conflict-exposure was assessed as having experienced an armed attack, abduction or injury during conflict. Respondents who were exposed to conflict were significantly more likely to tell someone (60%) about and seek help (40%) for IPV compared to those who were not directly exposed to conflict (*p* < 0.001) (Table 2).

**Table 2:** Conflict Exposure

	Told Someone	Sought Help
Non-Partner Sexual Violence (*n* = 779)		
Incident of SV occurred during a conflict event (conflict-related SV)	60% (55–64)***	36% (32–40)***
Intimate Partner Violence (*n* = 1163)		
Was exposed to conflict event (armed attack, abduction, injury)	60% (57–64) ***	40% (37–44)***

Note: *** p < 0.001

### Non-partner sexual violence: disclosure and help seeking behaviours

Respondents for whom the incident of sexual violence occurred during conflict had twice the odds of telling someone about their experience of violence, compared to those for whom the violence did not occur during conflict, after adjusting for other potential disclosure factors and socio-demographics (aOR: 2.2; 95%CI: 1.3–3.7). This relationship was statistically significant in the final adjusted model (*p* < 0.01). When examining other potential influences of violence disclosure, having a perpetrator who was an armed actor was also a significant factor associated with disclosure of NPSV in the final adjusted model (aOR: 1.9; 95%CI: 1.1–3.1; *p* < 0.05). In addition, the odds were 40% lower that respondents’ who agreed with the statement “*If a woman is raped, she has usually done something careless to put herself in that position”* would disclose their experience of violence compared to respondents who disagreed with this statement. This relationship was statistically significant in the final adjusted model (*p* < 0.05). In addition, older women (aged 30 and above) had about double the odds of disclosing non-partner violence they experienced compared to adolescents (aged 15–19) (women aged 30–39: aOR: 1.7; 95%CI: 1.1–2.8, *p* < .05; women aged 40–64: aOR: 2.1; 95%CI: 1.2–3.8, *p* < .01). See Table 3 for further details.

**Table 3:** Multivariate analysis for disclosure and help seeking for NPSV survivors

	Model 1: Multivariate analysis of factors associated with disclosure of NPSV		Model 2: Multivariate analysis of factors associated with help seeking after NPSV	
	Crude OR (95% CI)	aOR (95%CI)	Crude OR (95% CI)	aOR (95%CI)
Conflict-related Violence				
Had a direct conflict experience	1.7** (1.1–2.6)	2.2** (1.3–3.7)	3.7*** (2.2–6.2)	3.1*** (1.7–5.9)
Violence Disclosure Factors				
Type of Violence				
Other form of SV	1	1	1	1
Raped	1.1 (.7–1.5)	1.1 (.8–1.5)	1.1 (.7–1.6)	0.9 (.6–1.4)
Perpetrator of Violence				
Other person	1	1	1	1
Armed Actor/Police	1.6 (1.0–2.6)	1.9* (1.1–3.1)	1.9* (1.1–3.2)	1.4 (.8–2.5)
Agreed that a woman is to blame if she is raped	.7 (.5–1.1)	0.6* (.4–.9)	1.4 (.9–2.3)	1.1 (.7–1.9)
Socio-demographics				
Location				
Juba	1	1	1	1
Rumbek	1.8* (1.1–3.2)	1.6 (.8–3.0)	3.6** (1.7–7.9)	2.7* (1.1–6.8)
Juba PoCs	1.0 (.6–1.6)	0.7 (.3–1.6)	3.3** (1.5–7.7)	2.1 (.7–6.9)
Age				
15–19	1	1	1	1
20–29	1.2 (.8–1.8)	1.4 (.9–2.1)	1.2 (.7–1.9)	1.1 (.6–2.0)
30–39	1.5 (1.0–2.3)	1.7*(1.1–2.8)	1.3 (.7–2.3)	1.0 (.5–2.2)
40–64	2.0* (1.1–3.4)	2.1** (1.2–3.8)	2.1* (1.2–.3.9)	2.0 (1.0–4.0)
Income				
No income/humanitarian aid	1	1	1	1
Own work	1.9* (1.1–3.4)	1.6 (.8–3.2)	.8 (.4–.1.5)	0.7 (.3–1.5)
Husband	1.4 (.8–2.3)	1.3 (.7–2.3)	.9 (.5–1.6)	1.1 (.6–2.2)
Other	1.6 (1.0–2.6)	1.8* (1.0–3.2)	.7 (.4–1.2)	1.1 (.6–2.0)
Employment Status				
Not working	1	1	1	1
Student	1.1 (.7–1.8)	1.2 (.7–2.2)	.6 (.3–1.1)	.6 (.3–1.1)
Working	1.5 (1.0–2.3)	1.1 (.7–1.9)	1.3 (.8–2.1)	1.9* (1.1–3.2)
Fuel Source				
Leaves/grass/wood	1	1	1	1
Charcoal	1.2 (.8–1.8)	1.6 (1.0–2.5)	1.2 (.8–2.0)	2.0* (1.1–3.6)

Note: * p < 0.05, ** p < 0.01, *** p < 0.001; The final adjusted models were also controlled for the following socio-demographics which were not statistically significant in the final model: education, housing type and marital status. Standard errors were clustered at the boma/block level for all multivariate analysis

As with disclosure, experiencing conflict-related NPSV (versus non-conflict related violence) was associated with greater odds of seeking help (aOR: 3.1; 95%CI: 1.7–5.9, *p* < .001) in the final adjusted model. While the perpetrator being an armed actor was significantly associated with higher odds of accessing services at the bivariate level, after adjusting for further covariates it was no longer significant. None of the other potential violence factors examined were significantly associated with accessing services in the final adjusted model.

When examining socio-demographic factors in the final model, residents in Rumbek had more than two times the odds of accessing services compared to those resident in Juba (Rumbek- aOR: 2.7; 95%CI: 1.1–6.8). This relationship was statistically significant in the final adjusted model (*p* < 0.05). In addition, respondents who were working and used charcoal as their primary source of fuel had about twice the odds of accessing support services compared to those who were not working in the final model (work: aOR: l.9; 95%CI:1.1–3.2; *p* < .05; fuel: aOR: 2.0; 95%CI:1.1–3.6, *p* < .05). Table 3 for more information.

### Intimate partner violence: disclosure and help seeking behaviours

For survivors of IPV, conflict exposure (versus no conflict exposure) significantly increased the odds that a respondent would tell someone about her experience (aOR: 1.7; 95%CI 1.2–2.5; *p* < .01), after adjusting for other potential disclosure factors, martial circumstances and socio-demographics. When examining the potential influence of other violence disclosure factors, respondents who experienced physical and sexual violence (aOR: 2.2; 95%CI: 1.4–3.5) – had greater odds of telling someone about this violence than if they had experienced sexual violence alone. This relationship was significant even after adjusting for conflict exposure, other potential disclosure factors and social demographics (*p* < .001). Respondents also had greater odds of disclosing when the violence they experienced was more severe. For example, in the final model respondents who had been injured due to IPV (aOR: 2.7; 95%CI: 2.0–3.8), thought that the violence they experienced affected their wellbeing (a little effect: aOR: 1.7; 95%CI: 1.1–2.5; a large effect: aOR: 2.0; 95%CI: 1.2–3.3) and were afraid of their husbands some of the time (aOR: 1.5; 95%CI: 1.0–2.1) had greater odds of disclosing the violence. These relationships were significant after adjusting for all other factors (Injury: *p* < .001; Well-being: *p* < .01; Fear: *p* < .05).

When examining socio-demographic factors, the odds that respondents resident in the Juba PoCs would disclose an experience of IPV were 70% lower compared to those in Juba (aOR: 0.3; 95%CI: .1–.5). This relationship was statistically significant after adjusting for conflict exposure, potential violence disclosure factors and other socio-demographics in the final model (*p* < .001). See Table 4 for further details.

**Table 4:** Multivariate analysis of factors associated with disclosure of IPV

	Model 1: Multivariate analysis of factors associated with disclosure of IPV		Model 2: Multivariate analysis of factors associated with help seeking after IPV	
	Crude OR (95% CI)	AOR (95%CI)	Crude OR (95% CI)	AOR (95%CI)
Conflict-related Violence				
Had a direct conflict experience	1.8***(1.3–2.4)	1.7 ** (1.2–2.5)	2.0*** (1.3–3.0)	1.4 (.9–2.2)
Violence Disclosure Factors				
Type of Violence				
Sexual violence only	1	1	1	1
Physical violence only	2.4***(1.6–3.7)	1.5 (.9–2.5)	2.1* (1.1–3.9)	1.0 (.6–1.9)
Physical and sexual violence	4.9***(3.2–7.3)	2.2*** (1.4–3.5)	3.9*** (2.3–6.5)	1.7* (1.0–2.8)
Have experienced controlling behaviours	1.7* (1.1–2.4)	1.6* (1.1–2.4)	2.2*** (1.4–3.5)	2.2** (1.4–3.6)
Injured due to IPV	4.3*** (3.3–5.7)	2.7***(2.0–3.8)	4.6*** (3.2–6.6)	3.5***(2.4–5.2)
Effect on well-being due to IPV				
No effect	1	1	1	1
A little effect	3.1***(2.1–4.5)	1.7** (1.1–2.5)	2.5*** (1.5–4.1)	1.3 (.8–2.1)
A large effect	5.2*** (3.4–7.9)	2.0** (1.2–3.3)	3.3*** (1.9–5.9)	1.2 (.7–2.1)
Fear of Husband/Partner				
Never afraid	1	1	1	1
Afraid sometimes	2.5***(1.8–3.5)	1.5* (1.0–2.1)	1.3 (.9–2.0)	.6* (.4–1.0)
Afraid most of the time	4.2*** (2.7–6.7)	1.7 (1.0–2.8)	2.0* (1.2–3.4)	.7 (.4–1.2)
Agreed that violence from husband against wife was justified in at least 1 occasion	1.1* (1.0–1.3)	1.0 (.9–1.2)	1.3*** (1.2–1.5)	1.1 (1.0–1.3)
Marital Characteristics				
Bride price paid upon marriage	1.6** (1.2–2.2)	1.1 (.7–1.6)	2.4*** (1.6–3.5)	1.3 (.8–2.1)
Marriage was forced	1.9***(1.4–2.7)	1.3 (.9–2.0)	2.2*** (1.5–3.1)	1.5* (1.0–2.3)
Socio-demographics				
Location				
Juba	1	1	1	1
Rumbek	2.5***(1.6–3.8)	1.3 (.8–2.2)	6.2*** (3.7–10.3)	3.2*** (1.8–5.8)
Juba PoCs	.8 (.5–1.1)	.3*** (.1–.5)	2.3** (1.4–3.9)	.9 (.4–1.8)

Note: * p < 0.05, ** p < 0.01, *** p < 0.001; The final adjusted models were also controlled for the following socio-demographics which were not statistically significant in the final model: age, source of income, employment status, education, fuel source, housing type and marital status. Standard errors were clustered at the boma/block level for all multivariate analysis

As with disclosure of IPV, conflict exposure significantly increased the odds that a respondent would seek support services (OR: 2.0; 95%CI: 1.3–3.0; *p* < .001), however this relationship was no longer significant after adjusting for other factors in the final model. When examining the potential influence of other violence disclosure factors, respondents who experienced physical and sexual violence (aOR: 1.7; 95%CI: 1.0–2.8; *p* < .05) had greater odds of telling someone about this violence than if they had experienced sexual violence alone. Respondents also had increased odds of disclosing violence if they had experienced controlling behaviours (aOR: 2.2; 95%CI: 1.4–3.6; *p* < .01), if their marriage was forced (aOR:1.5; 95%CI:1.0–2.3; *p* < .05) and if they were injured (aOR: 3.5; 95% CI: 2.4–5.2, *p* < .001), after controlling for other factors. Respondents had lower odds of seeking services if they reported they were sometimes afraid of their husbands, compared to never being afraid of them (aOR: .6; 95%CI: .4–1.0; *p* < .05). See Table 4 for further details.

Secondary analysis examined if there were any differences amongst respondents accessing formal versus informal or community-based services. Conflict-exposure was associated with accessing formal services for survivors of NPSV (aOR: 3.7; 95%CI:1.0–13.3; *p* < .05) but not for those experiencing IPV. For survivors of IPV, physical violence (compared to sexual violence) and income from a husband or other source (versus no income/dependence on humanitarian aid) increased the odds of accessing formal services (violence: aOR 3.6; 95%CI:1.2–10.6; *p* < .05; income: from husband: aOR: 3.2; 95%CI: 1.5–7.0; *p* < .01; from other source: aOR: 2.4; 95%CI: 1.1–5.1; *p* < .05). Women and girls who reported they experienced controlling behaviours from their partner or were in a forced marriage had decreased odds of accessing formal services (behaviours: aOR: .3; 95%CI:.1–.9; *p* < .05; marriage: aOR: .5; 95%CI: .3–1.0; *p* < .05). For survivors of NPSV, in addition to conflict-exposure, working (aOR:3.6; 95%CI:1.0–13.1; *p* < .05), using charcoal as fuel source (aOR: 3.3; 95%CI:1.2–9.4; *p* < .05) and having ever been married (aOR: 3.4; 95%CI: 1.3–8.9; *p* < .05) were associated with increased odds of accessing formal services. See Table 5 for more information.

**Table 5:** Multivariate analysis of factors associated with formal versus informal/community-based services, among women who accessed any service

	Model 1: Multivariate analysis of factors associated with formal services for IPV (*n* = 414)		Model 2: Multivariate analysis of factors associated with formal services for NPSV (*n* = 205)	
	Crude OR (95% CI)	AOR (95%CI)	Crude OR (95% CI)	AOR (95%CI)
Conflict-related Violence				
Had a direct conflict experience	.6 (.3–1.3)	.6 (.3–1.3)	2.7* (1.0–6.9)	3.7* (1.0–13.3)
Violence Disclosure Factors				
Type of Violence				
(IPV) Sexual violence only/(NPSV) Other form of SV	1	1	1	1
(IPV) Physical violence only/(NPSV) Raped	2.7(.9–7.5)	3.6 * (1.2–10.6)	1.2 (.5–2.6)	1.3 (.6–3.3)
(IPV) Physical and sexual violence	2.4 (.9–6.8)	3.0* (1.0–8.5)	–	–
Have experienced controlling behaviours	.4* (.2–.9)	.3* (.1–.9)	–	–
Marital Characteristics				
Marriage was forced	.8 (.4–1.4)	.5* (.3–1.0)	–	–
Socio-demographics				
Income				
No income/humanitarian aid	1	1	1	1
Own work	1.0 (.5–2.0)	1.1 (.5–2.4)	.3 (.1–1.0)	.4 (.1–2.4)
Husband	1.8 (.9–3.5)	3.2** (1.5–7.0)	.5 (.2–1.5)	1.0 (.2–5.9)
Other	1.4 (.7–2.7)	2.4* (1.1–5.1)	.4 (.1–1.0)	1.7 (.4–6.9)
Employment Status				
Not working	1	1	1	1
Student	.4*(.2–1.0)	.4 (.1–1.3)	.2** (.1–.6)	.2** (0–.6)
Working	2.2* (1.3–3.9)	2.4* (1.2–5.1)	1.6 (.7–3.6)	3.6* (1–13.1)
Fuel Source				
Leaves/grass/wood	1	1	1	1
Charcoal	.8 (.4–1.4)	.8 (.4–1.4)	2.1 (1.0–4.8)	3.3* (1.2–9.4)
Ever Married	3.1 (.6–15.1)	3.2 (.8–12.7)	2.3* (1.2–4.6)	3.4* (1.3–8.9)

Note: * p < 0.05, ** p < 0.01; The final adjusted models were also controlled for the following socio-demographics in the final model: location, age, education, and housing type. The IPV model also included variables to account for fear of husband/partner, effect of IPV on wellbeing, injury, payment of brideprice and justification of violence which were not statistically significant. The NPSV model also included variables about the perpetrator of violence and attitudes towards rape which were not statistically significant. Standard errors were clustered at the boma/block level for all multivariate analysis

## Discussion

Exposure to armed conflict is associated with disclosure and help seeking behaviours among survivors of VAWG in South Sudan. For NPSV, survivors who had experienced SV during a conflict-incident had greater odds of telling someone about their experience as well as seeking help. This finding was consistent with secondary analysis where women who experienced conflict-related SV had greater odds of accessing formal versus informal/community-based services. This is an important finding for the international community as, given the stigma associated with experiences of NPSV in this context, it was not known whether those who experienced conflict-related SV would be more or less likely to tell someone or report to support services.

For respondents who had experienced IPV, conflict-exposure also increased the odds of telling someone about the experience, though it was not associated with a woman or girl’s likelihood of seeking help. Importantly there was more variation of help seeking behaviour by site with respondents from the PoCs less likely to tell someone about their experiences of IPV compared to respondents in Juba. This difference could be due to potential long-term impacts of trauma associated with exposure to conflict that remains unaddressed in the PoCs, as well as weakened social networks and increased economic insecurity of residents in the displacement sites. Furthermore, PoC residents may have been less inclined to disclose the violence they were experiencing in their homes as they would be unable to leave the PoCs in order to separate themselves from their abusive partner.

Armed conflict was not the only factor that influenced disclosure and help seeking in this context. Internalized patriarchal norms and agreement that violence is acceptable also had an effect on these behaviours. For NPSV, respondents who held the inequitable view that *“women who were raped often put themselves in that position”* had decreased odds of telling someone about their experience of violence, however agreement with this statement did not have an association with seeking help. Furthermore, for survivors of IPV, respondents who agreed that violence perpetrated by a husband against his wife could be justified in at least one circumstance had increased odds of reporting and seeking help until the model was adjusted for other factors and this relationship became non-significant. This finding was potentially influenced by respondents from Rumbek – where women held particularly inequitable views about gender equality and also had higher reporting/disclosure rates compared to the other two sites violence [18]. Overall, these findings suggest that while attitudes and beliefs about the acceptability of VAWG may have some influence on disclosure and help seeking, they are not as important as other factors such as exposure to armed conflict.

The severity of IPV also appeared to have an impact on disclosure and help seeking behaviours. Women and girls had increased odds of telling someone about their experience of violence if they had been injured, if the violence affected their well-being, if they were sometimes afraid of their partners, and if they experienced controlling behaviours. Many of these factors were also important for respondents who sought to access support services. Injury appeared to have the greatest impact on help seeking behaviours for survivors of IPV with respondents who reported an injury having more than 3 times the odds of accessing services compared to those who were never injured.

Interestingly women who reported that were controlled by their partners had twice the odds of accessing services, indicating that they were able to circumvent these behaviours to get help. However, secondary analysis showed that women were more likely to access informal or community-based services, rather than formal help. Women who were sometimes afraid of their partner some of the time were less likely to access services and there was no relationship between access to services and two measures of severity - being afraid of one’s partner all of the time and self-reported impact of IPV on well-being. These findings suggest that while women and girls experiencing more severe violence may be more likely to tell someone about their experiences, this does not necessarily lead to accessing support services. The international community needs to continue to examine pathways to help women and girls who disclose to an informal source (such as a friend or family member), given the wide gap between disclosure and help seeking rates for survivors of IPV.

Further characteristics such as age may also impact help seeking behaviours. For NPSV, older women were more likely to disclose their experience of violence though this did not affect their access to support services. Conversely, age was not significantly associated with disclosure or help seeking behaviours among women and girls experiencing IPV. Given the potential that stigma and the loss of a girl’s virginity may impact her marriageability in this context, it is likely that the consequences of disclosure for girls and younger women would be more severe compared to older women in the community, which could explain this trend.

Finally, poverty and women’s economic empowerment also were associated with disclosure and help seeking for survivors of violence. For example, women who were working had almost twice the odds of accessing support services after an incident of NPSV. Potential barriers to service access for women and girls in this setting include the costs associated with services as well as the freedom of movement to leave the house to get to service locations. This finding shows that, at least for NPSV, women who are working have some increased ability to access services. This finding was echoed in secondary analysis of the IPV data where women who were accessing services had greater odds of accessing formal rather than informal/community-based services if they were working.

### Limitations

There were a number of limitations to this analysis. For one, this analysis was done on a wider survey that had been developed to understand prevalence of VAWG rather than disclosure and help seeking behaviours. As such, some relevant variables, such as awareness of services, were not collected in the original survey and thus could not be included in the analysis. In addition, all data were collected via self-report and, given the sensitive subject matter, could be subject to disclosure bias. The analysis was also conducted cross-sectional data and, as such, it explores factors that are associated with disclosure and help-seeking, rather than factors that cause such behaviours. Finally, the definition of support services was quite broad and encompassed multiple services that ranged from medical and legal support to local chiefs/traditional leaders and courts. Secondary analysis was conducted examining reporting to formal versus informal/community-based structures but the sample – particularly for NPSV – was somewhat small. There could be differing enablers and barriers to accessing differing forms of support that, by combining these services together into these summary variables, this analysis was unable to examine. However, due to the small sample size of respondents who accessed support services, further sub-analysis by specific type of service was not possible.

## Conclusions

These findings are important as, prior to this analysis, it was unclear how experiencing conflict-related VAWG would affect disclosure and help seeking behaviours. This analysis clearly shows that conflict-related violence is more likely to be disclosed and those who experience this type of violence are more likely to access to support services. Given the findings of this paper, it is important that the international community work to reduce barriers to reporting/help seeking for non-conflict-related forms of violence in these settings.

## Data Availability

Survey materials are available by request to the corresponding author.

## Abbreviations

GWI: Global Women’s Institute
IPV: Intimate partner violence
IRB: Institutional Review Board
IRC: International Rescue Committee
NPSV: Non-partner sexual violence
PoC: Protection of Civilian
SV: Sexual Violence
UN: United Nations
VAWG: Violence against women and girls
